# Supplementary material for: A Likelihood Approach for Real-Time Calibration of Stochastic Compartmental Epidemic Models
Source: PLoS Comput Biol. 2017 Jan 17;13(1):e1005257. doi: 10.1371/journal.pcbi.1005257 (PMC5240920; doi:10.1371/journal.pcbi.1005257)
Supplement: S1 File — (TAR.GZ) [file pcbi.1005257.s014.tar.gz › HSPH_Online-SI-Revision/IPoi09/n1-extreme/n1-extreme_BENCH2_summary.pdf]

peak -8	3w spec	3w cum	5w spec	5w cum	end	3w spec LOG	3w cum LOG	5w spec LOG	5w cum LOG	end LOG
mean	133.5	146.3	80.1	98.1	40.	0.8	0.8	1.	0.9	0.9
median	95.3	98.8	77.9	82.7	39.3	0.8	0.7	0.9	0.7	0.6
quantile .1	58.	57.3	39.6	48.	14.8	0.3	0.3	0.3	0.3	0.1
quantile .9	207.4	182.3	109.2	127.5	64.	1.4	1.2	1.7	0.3	1.9
44	45	35	44	26						

peak -4	3w spec	3w cum	5w spec	5w cum	end	3w spec LOG	3w cum LOG	5w spec LOG	5w cum LOG	end LOG
mean	37.1	42.7	38.5	32.	23.5	0.3	0.3	0.3	0.2	0.2
median	32.7	40.4	33.7	28.4	19.3	0.2	0.2	0.2	0.2	0.1
quantile .1	19.	18.7	22.4	16.5	8.7	0.1	0.1	0.1	0.1	0.
quantile .9	54.7	67.5	54.5	48.2	39.3	0.5	0.4	0.7	0.1	0.4
41	41	35	37	41						

peak	3w spec	3w cum	5w spec	5w cum	end	3w spec LOG	3w cum LOG	5w spec LOG	5w cum LOG	end LOG
mean	37.2	30.3	47.9	31.9	36.9	0.2	0.2	0.3	0.2	0.3
median	33.7	28.1	42.8	28.7	32.6	0.2	0.1	0.2	0.1	0.2
quantile .1	24.6	18.7	31.8	20.4	24.1	0.1	0.1	0.1	0.1	0.1
quantile .9	49.3	41.3	67.2	42.4	51.3	0.3	0.2	0.4	0.1	0.3
45	40	41	44	43						

peak +4	3w spec	3w cum	5w spec	5w cum	end	3w spec LOG	3w cum LOG	5w spec LOG	5w cum LOG	end LOG
mean	82.5	53.9	91.2	58.3	65.9	0.3	0.3	0.4	0.3	0.3
median	54.1	46.9	66.	48.2	51.	0.2	0.2	0.3	0.2	0.2
quantile .1	37.5	34.8	45.4	36.	39.8	0.2	0.1	0.2	0.1	0.2
quantile .9	142.8	72.1	155.9	76.8	100.	0.4	0.3	0.5	0.1	0.3
42	43	42	44	42						

peak +8	3w spec	3w cum	5w spec	5w cum	end	3w spec LOG	3w cum LOG	5w spec LOG	5w cum LOG	end LOG
mean	132.5	83.3	552.3	91.3	100.9	0.4	0.3	0.5	0.3	0.4
median	69.	64.6	80.5	65.2	71.7	0.3	0.2	0.4	0.2	0.2
quantile .1	53.6	48.	57.1	48.	49.1	0.2	0.2	0.3	0.2	0.2
quantile .9	192.6	132.	755.3	158.6	171.7	0.7	0.4	1.2	0.2	0.5
42	42	45	39	40	{}	{}	{}	{}	{}	{}
